# Combined effects of interleukin-1β and cyclic stretching on metalloproteinase expression in corneal fibroblasts in vitro

**DOI:** 10.1186/s12938-016-0198-6

**Published:** 2016-06-10

**Authors:** Pengfei Feng, Xiaona Li, Weiyi Chen, Chengxing Liu, Shuo Rong, Xiaojun Wang, Genlai Du

**Affiliations:** Institute of Applied Mechanics and Biomedical Engineering, Taiyuan University of Technology, Taiyuan, 030024 China; College of Mechanics, Taiyuan University of Technology, Taiyuan, 030024 China; Biology Department, Taiyuan Normal University, Jinzhong, 030619 China

**Keywords:** Cyclic stretching, Interleukin-1β, Corneal fibroblasts, Matrix metalloproteinases

## Abstract

**Background:**

Corneal tensile strain increases if the cornea becomes thin or if intraocular pressure increases. However, the effects of mechanical stress on extracellular matrix (ECM) remodelling in the corneal repair process and the corneal anomalies are unknown.

**Methods:**

In this study, the combined effects of interleukin-1β (IL-1β) on matrix metalloproteinases (MMPs) in corneal fibroblasts under cyclic stretching were investigated in vitro. Cultured rabbit corneal fibroblasts were subjected to 5, 10 or 15 % cyclic equibiaxial stretching at 0.1 Hz for 36 h in the presence of IL-1β. Conditioned medium was harvested for the analysis of MMP2 and MMP9 protein production using the gelatin zymography and western blot techniques.

**Results and conclusions:**

Cyclic equibiaxial stretching changed the cell morphology by increasing the contractility of F-actin fibres. IL-1β alone induced the expression of MMP9 and increased the production of MMP2, and 5 % stretching alone decreased the production of MMP2, which indicates that a low stretching magnitude can reduce ECM degradation. In the presence of IL-1β, 5 and 10 % stretching increased the production of MMP2, whereas 15 % stretching increased the production of MMP9. These results indicate that MMP expression is enhanced by cyclic mechanical stimulation in the presence of IL-1β, which is expected to contribute to corneal ECM degradation, leading to the development of post-refractive surgery keratectasia.

## Background

Corneal refractive surgery is one of the most widely used methods for the correction of refractive errors [1]. The distribution of stress or strain in the cornea under intraocular pressure (IOP) was investigated. Animal experiments [2–4] and finite element simulation [5, 6] show that corneal membrane stress or strain increases after keratorefractive surgery. As a load-bearing tissue, the cornea is mainly subjected to biaxial tensile stress because of IOP in vivo [7]. IOP fluctuates during the occurrence of hypertension and the change in head positions or sleeping postures [8–10]. The combination of tight eye closure and forceful rubbing may increase the IOP to more than 10 times the normal levels, perhaps even higher [11]. The same type of eye rubbing activity following keratorefractive surgery may have a greater chance of causing an adverse response, including ectasia, in a thinner or otherwise more susceptible cornea [11].

Interleukin-1β (IL-1β), a proinflammatory cytokine, is constitutively expressed in the corneal epithelium [12]. After injury, IL-1β is released from the epithelium into the tear and the corneal stroma as a master modulator of the corneal wound healing cascade [13]. Proinflammatory cytokines are also involved in the pathogenesis of dry eye disease [14, 15], which is one of the most common complications after keratorefractive surgery [16].

Matrix metalloproteinases (MMPs) are a family of proteinases that initiate the degradation of collagen and other extracellular matrix components [17]. Two gelatinases, namely, 72 kDa gelatinase A (MMP2) and 92 kDa gelatinase B (MMP9), are the primary matrix-degrading enzymes that are initially secreted as proenzymes (Pro-MMP2 and Pro-MMP9) [18]. MMP2 and MMP9 play important roles in corneal wound healing after refractive surgery [19, 20], corneal ulceration [21], post-refractive surgery keratectasia [22–24], and the pathogenesis of dry eye disease [14].

In vivo, most living cells are exposed to a variety of biomechanical forces [25]. Mechanical stimulation is involved in the regulation of MMPs in some ocular tissues, such as sclera [17, 26, 27], trabecular meshwork [28] and lamina cribrosa cells [29]. Corneal fibroblasts have been demonstrated to respond actively to local tension changes in the ECM [30], and fibroblasts are a major type of mechanoresponsive cell [31]. Our previous work showed that large-magnitude stretching alone increased the protein expression of MMP2 in rabbit corneal fibroblasts at 24 h in an ERK-dependent manner [32].

We speculate that the increased strain caused by thinning of the cornea or the abnormally high IOP induces changes in ECM metabolism and alters the biomechanical properties of the cornea under the action of proinflammatory cytokines. In this study, we will focus on the combined effects of cyclic stretching and IL-1β on the production of MMP2 and MMP9 by rabbit corneal fibroblasts to investigate the role of mechanical stimulation in corneal ECM synthesis.

## Methods

### Preparation of corneal fibroblasts

New Zealand White rabbits were obtained from Shanxi Medical University (n = 6). Animals were anaesthetised with ether and killed using the air embolism method. Corneal keratocytes were immediately isolated and maintained as described previously [33]. In brief, the epithelial sheet and endothelial layer of the cornea were removed mechanically and the remaining tissue was treated with type II collagenase (2 mg/mL, in DMEM/F12) (Thermo Fisher Scientific, USA) at 37 °C until a single-cell suspension was obtained. Isolated corneal keratocytes were cultured in DMEM/F12 medium supplemented with 10 % foetal bovine serum (FBS; Gibco, USA) and maintained at 37 °C in a humidified atmosphere of 5 % CO_2_ in air (cultured keratocytes will be referred to as corneal fibroblasts if they are cultured in serum) [12]. All of the experiments were conducted on cells between the third and fifth passage.

### Cyclic equibiaxial stretching and IL-1β treatment on corneal fibroblasts

Corneal fibroblasts were seeded on six-well Bioflex^®^ plates (Flexcell Int. Corp., Hillsborough, NC, USA) with flexible bottoms coated with type I collagen. When the cell confluency reached 70 %, the medium was removed and replaced with DMEM/F12 medium containing 1 % FBS and recombinant human IL-1β (0, 0.2, 0.4 and 0.8 ng/mL). Then, the cells were subjected to a mechanical strain of downward deformation by a computer-controlled vacuum using a Flexcell^®^ Tension Plus™ FX-4000™ system (Flexcell Int. Corp., Hillsborough, NC, USA) with an elongation rate of 5, 10 or 15 % that was alternatively applied (0.1 Hz, 36 h, sine waveform).

The cell culture supernatants were collected at 36 h as protein samples for zymography or western blot analysis. The protein concentration was measured using BCA kit (Applygen Technologies Inc., Beijing, China).

### Fluorescence staining and confocal microscopy

After 36 h of cyclic equibiaxial stretching (5, 10 and 15 % at 0.1 Hz), cells were washed twice in phosphate-buffered saline (PBS) and fixed in 4 % paraformaldehyde for 30 min. Then, the cells were permeabilised for another 10 min with 0.5 % Triton-X 100 in PBS. Cells were incubated with rhodamine B (Sigma, USA) for 30 min to stain the F-actin filaments. The cell nucleus was stained with 4′, 6-diamidino-2-phenylindole (DAPI) diluted in PBS 1:20 for 10 min. Finally, fluorescence images were collected using an Olympus FV1000 confocal microscope.

### Gelatin zymography

Gelatin zymography was used to analyse the relative concentrations of MMP2 and MMP9 secreted into the cell culture medium using a previously reported method [34]. In brief, each protein sample containing the same quantity of protein (10 µg) was separated by 8 % SDS-PAGE containing 0.1 % gelatin. The gels were washed in twice 2.5 % Triton-X 100 for 45 min and incubated in renaturing buffer at 37 °C for 42 h. The gels were stained with 0.05 % coomassie blue for approximately 2 h and were destained until bands were clearly visible. The gels were scanned, and the band intensities were quantitated using the Image Pro Plus 5.1 software (Medium Cybernetics Inc., Bethesda, MD, USA).

### Western blot

Pre-stained markers and protein samples with equal amounts of total protein were separated by electrophoresis on 8 % SDS-PAGE gels and transferred to polyvinylidene difluoride membranes (Millipore, USA). The membranes were blocked with TBST containing 5 % non-fat milk. The procedure was followed by overnight incubation with a monoclonal antibody to MMP2 (1:1000) or MMP9 (1:500; Abcam, UK) at 4 °C, six washes with 0.01 % Tween-TBS buffer, and a 2 h incubation with a secondary antibody (anti-mouse HRP-linked, 1:7000; Abcam, UK). GAPDH (1:100; Boster, China) was used as control for equal protein loading. The band intensities were quantitated using the Image Pro Plus 5.1 software.

### Statistical analysis

All of the data from three independent experiments are presented as the mean ± standard deviation. Statistical analysis was conduted using one-way ANOVA followed by a post hoc Tukey’s test or using a Student’s *t* test to compare the experiments between only two groups. A value of p < 0.05 was considered significant.

## Results

### Cyclic equibiaxial stretching changed the cell morphology of corneal fibroblasts

**Fig. 1:**
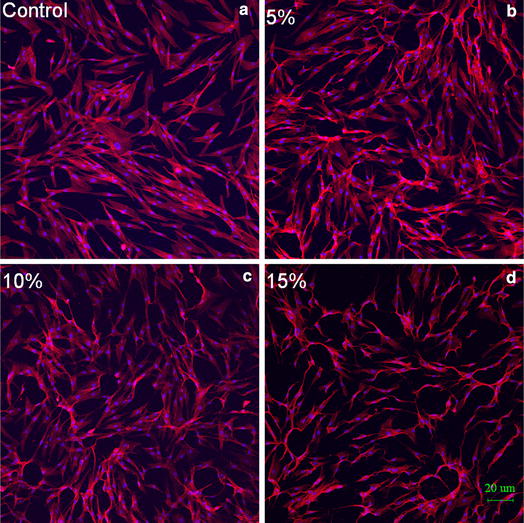
The effects of cyclic stretching on cell morphology are shown. F-actin and nuclei of the cells were stained with rhodamine B and DAPI. **a** The cells were in the static condition. **b**–**d** The cells were exposed to cyclic stretching for 5, 10 and 15 % respectively. *Red* F-actin; *Blue* cell nuclei

After cyclic equibiaxial stretching, F-actin and nuclei were stained with rhodamine B and DAPI, respectively, to analyse the cell morphology. Confocal microscopy of actin filaments showed that cells under stretching or non-stretching had a random fibre orientation and had rounded cell bodies with extensions in multiple directions. Cyclic stretching increased the contractility of F-actin fibres at large magnitude. The effects of cyclic stretching on the alignment of actin fibres are shown in Fig. 1.

### IL-1β increased Pro-MMP2 and induced Pro-MMP9 secretion by corneal fibroblasts

**Fig. 2:**
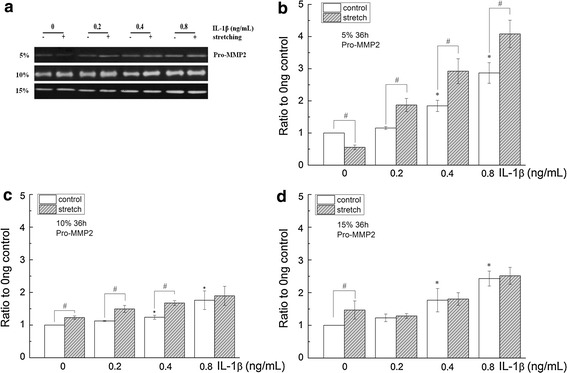
The effects of cyclic stretching on MMP2 activity in corneal fibroblasts treated with IL-1β. **a** Zymographic analysis of the conditioned medium treated with IL-1β (0, 0.2, 0.4, or 0.8 ng/mL) for 36 h with or without stretching (5, 10 or 15 %). **b**–**d** The indicated quantitative data refer to the control and IL-1β groups after cyclic stretching for 36 h and are the mean of three different experiments (n = 6). *p < 0.05, ratio compared to 0 ng/mL IL-1β; ^#^p < 0.05, compared with the static control at the same IL-1β treatment

Pro-MMP2 was detected in the supernatant of cells with different treatments (0, 0.2, 0.4 and 0.8 ng/mL). Pro-MMP2 produced by cells treated with 0.4 and 0.8 ng/mL IL-1β are significantly higher (p = 0.002 and p = 0.000, respectively) than cells treated with 0 ng/mL IL-1β (Fig. 2*). However, Pro-MMP9 was undetectable in the conditioned medium harvested from cultures in the absence of IL-1β. IL-1β induced the expression of Pro-MMP9 and increased the production of Pro-MMP9 at 0.4 or 0.8 ng/mL compared with 0.2 ng/mL (p = 0.033 and p = 0.000, respectively) (Fig. 4*).

### Cyclic stretching alone had a bidirectional effect on Pro-MMP2 and further increased Pro-MMP2 and Pro-MMP9 in the presence of IL-1β

**Fig. 3:**
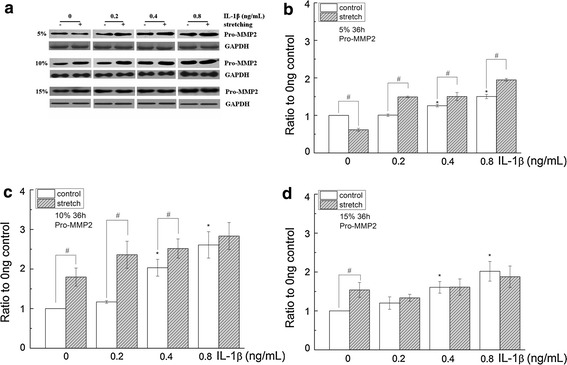
The effects of cyclic stretching and IL-1β on MMP2 activity in corneal fibroblasts. MMP2 was detected by western blot (**a**) and quantified by densitometry (**b**–**d**). The indicated quantitative data are the mean of three different experiments (n = 6). *p < 0.05, ratio compared to 0 ng/mL IL-1β; ^#^p < 0.05, compared with the static control at the same IL-1β treatment

After 36 h of cyclic equibiaxial stretching, Pro-MMP2 and Pro-MMP9 in the cell supernatant were determined. Statistical analysis of Pro-MMP2 and Pro-MMP9 was conducted to compare the ‘stretched’ and ‘non-stretched’ groups (static control). The results showed that 5 % stretching decreased the expression of Pro-MMP2 (p = 0.03), whereas 10 and 15 % stretching increased the expression of Pro-MMP2 in the absence of IL-1β (Figs. 2, 3). In the presence of IL-1β, 5 % stretching exhibited a significant increase in the expression of Pro-MMP2 compared with the corresponding static control. With 10 % cyclic stretching, Pro-MMP2 was statistically increased at 0.2 and 0.4 ng/mL, but not at 0.8 ng/mL IL-1β treatment. Compared with the static control, 15 % stretching did not significantly change the expression of Pro-MMP2 by corneal fibroblasts receiving IL-1β treatment (Figs. 2, 3).

**Fig. 4:**
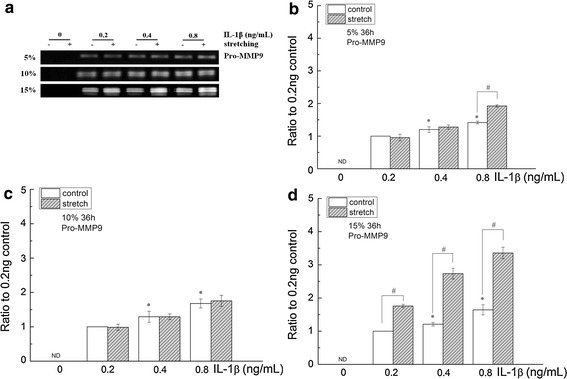
The effects of cyclic stretching on MMP9 activity in corneal fibroblasts treated with IL-1β. **a** Zymographic analysis of the conditioned medium treated with IL-1β (0, 0.2, 0.4 or 0.8 ng/mL) for 36 h with or without stretching (5, 10 or 15 %). **b**–**d** The indicated quantitative data refer to the control and IL-1β groups after cyclic stretching for 36 h and are the mean of three different experiments (n = 6). *ND* not detected; *p < 0.05, ratio compared to  0.2  ng/mL IL-1β; ^#^p < 0.05, compared with the static control at the same IL-1β treatment

**Fig. 5:**
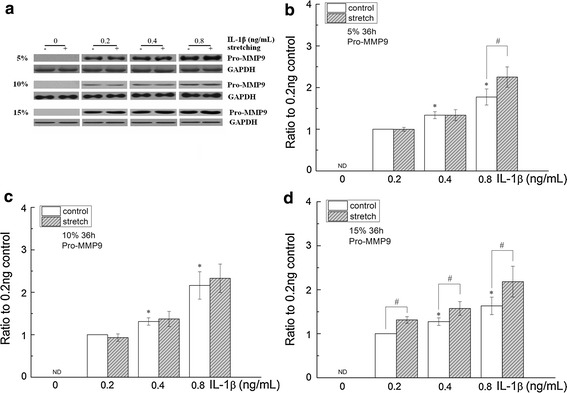
The effects of cyclic stretching and IL-1β on MMP9 activity in corneal fibroblasts. MMP9 was detected by western blot (**a**) and quantified by densitometry (**b**–**d**). The indicated quantitative data are the mean of three different experiments (n = 6). ND, not detected; *p < 0.05, ratio compared to  0.2 ng/mL IL-1β; ^#^p < 0.05, compared with the static control at the same IL-1β treatment

In the absence of IL-1β, Pro-MMP9 was still undetectable under cyclic stretching with any of the three elongation rates (Figs. 4, 5). In the presence of IL-1β, the expression of Pro-MMP9 was significantly upregulated in the 15 % stretching group (0.2, 0.4 and 0.8 ng/mL IL-1β treatment) and the 5 % stretching group (0.8 ng/mL IL-1β treatment). By contrast, this upregulation was not observed in all of the 10 % stretching groups.

All of the aforementioned results were highly concordant between gelatin zymography and western blot analyses.

## Discussion

Corneal fibroblasts experience complex mechanical strains with myriad amplitudes and frequencies after keratorefractive surgery. We attempted to duplicate the conditions of the physical and chemical environments of corneal fibroblasts in the post-refractive surgery cornea or the cornea under high IOP conditions (such as abnormal eye rubbing) to understand the effects of various types of mechanical stimulation on ECM synthesis. The parameters used in this experiment were 5, 10 and 15 % biaxial stretching at 0.1 Hz maintained for 36 h, which have been used by other investigators using other cell types [17, 35, 36]. If the cornea is treated as a simple pressure vessel, then the strain across the cornea will be directly proportional to IOP and inversely proportional to the modulus and thickness of the cornea [3, 37]. Notably, the 5, 10 and 15 % strain used in our experiments could emulate the effect of the increase in IOP or the decrease in the modulus and thickness of the cornea.

In the absence of IL-1β, 5 % stretching decreased the expression of Pro-MMP2, whereas 10 and 15 % stretching increased the expression of Pro-MMP2; similar results have been observed in human patellar tendon fibroblasts [38]. The major finding of this study is that cyclic equibiaxial stretching did not change the cell orientation but changed the cell morphology by increase the contractility of F-actin fibres. IL-1β alone induced the expression of Pro-MMP9 and increased the expression of Pro-MMP2, which were further enhanced by 15 % stretching or 5 and 10 % stretching, respectively; In the presence of IL-1β, rabbit corneal fibroblasts exhibited different responses to the distinctive strain patterns. All of these findings, together with the findings of this study, indicate that IL-1β combined with mechanical strain may lead to tissue destruction under some conditions.

Mechanical stretching has been shown to regulate MMPs in other types of ocular cells [17, 26, 27, 29]. A 15 % cyclic equibiaxial stretching resulted in a significant increase in Pro-MMP2 after 12 and 48 h and significantly increased the production of the active form of MMP2 after 48 h. Increased levels of active MMP2 in the sclera would be expected to contribute to scleral ECM degradation, scleral thinning and possible ocular ectasia [17]. In this study, 15 % cyclic stretching and IL-1β had no synergistic effect on the protein level of MMP2. This finding may be due to the following reasons. First, unlike MMP9, which was undetectable in the absence of IL-1β, MMP2 was constitutively produced by corneal fibroblasts at a level that can be easily visualised by zymography or Western blot [39]. This finding indicated that MMP2 may serve a daily surveillance function [40] and be involved in long-term regulation during the corneal ECM remodelling process, as other researchers have indicated [41]. Second, IL-1β combined with cyclic stretching may alter the pattern of MMP expression by the corneal fibroblasts.

Type IV collagen and type V collagen are distributed in the basement membrane of the corneal epithelium and the corneal stroma, which provide the majority of the tensile strength of the cornea [7]. The destruction of collagen fibrils will lead to corneal thinning and abnormalities in the structure of the cornea and weaken the biomechanical strength of the cornea. This study has shown that the combination of IL-1β and cyclic stretching (5 and 10 %) increases MMP2 expression and that 15 % stretching amplifies IL-1β-induced MMP9. These MMPs are directly involved in collagen degradation, which may explain why high distending forces located on the thinner or weakened cone apex (e.g., forceful eye rubbing) tend to promote the development or progression of some forms of post-refractive surgery keratectasia, particularly with inflammation.

Living connective tissue is designed to combat mechanical stress without failing. However, corneal repair mechanisms may be different from those in other tissues with functions that depend on form and strength but not transparency [42]. Several studies have shown that overload induces MMPs and participates in ECM degradation [17]. Notably, static and dynamic fatigue processes, particularly dynamic fatigue, hasten mechanical failure and enzymatic proteolysis occurs more rapidly in bovine pericardium subjected to fatigue [43]. The development of post-refractive surgery keratectasia is a complicated and multifactorial process, and the studies on the effects of mechanical stretching and inflammatory mediators on corneal fibroblasts are likely simplistic approximations of the dynamic environment in which corneal fibroblasts reside. However, the present study demonstrates that corneal fibroblasts can respond to mechanical load and that tensile stress can amplify IL-1β-induced MMP expression. Corneal stromal cells reside within a complex 3-D matrix in vivo, and MMPs play an important role in mediating corneal stromal cell spreading, migration [44], and ECM remodelling [33] within the 3-D collagen matrix. The potential effects of mechanical stretching on the expression of MMPs by corneal stromal cells in 3-D matrix remodelling should be investigated further.

## Conclusions

Corneal fibroblasts were subjected to combined cyclic stretching and IL-1β to determine the effect of mechanical strain on corneal ECM metabolism. The activities of MMP2 and MMP9 were evaluated. The results of the experiment indicated that a low stretching magnitude (5 %) can reduce corneal ECM degradation, whereas large stretching magnitudes (10 and 15 %) can increase the degradation of the corneal ECM in the absence of IL-1β. IL-1β alone induced the expression of Pro-MMP9 and increased the expression of Pro-MMP2. In the presence of IL-1β, the expression of MMPs was further enhanced by mechanical stretching, and the increased MMPs would be expected to participate in the corneal ECM remodelling, ultimately leading to post-refractive surgery keratectasia. The study confirmed the hypothesis that mechanical stretching together with IL-1β participate in MMP regulation and ECM remodelling in corneal fibroblasts, which indicate the importance of controlling the inflammatory response and avoiding the eye rubbing activity following a corneal refractive surgery procedure.

## Abbreviations

IOP: intraocular pressure
IL-1β: interleukin-1β
ECM: extracellular matrix
MMPs: matrix metalloproteinases
MMP2: matrix metalloproteinase 2
MMP9: matrix metalloproteinase 9
Pro-MMP2: the precursor of matrix metalloproteinase 2
Pro-MMP9: the precursor of matrix metalloproteinase 9
ERK: extracellular signal-regulated kinase
